# Assessment of Adherence to the Core Elements of Hospital Antibiotic Stewardship Programs: A Survey of the Tertiary Care Hospitals in Punjab, Pakistan

**DOI:** 10.3390/antibiotics10080906

**Published:** 2021-07-24

**Authors:** Naeem Mubarak, Asma Sarwar Khan, Taheer Zahid, Umm e Barirah Ijaz, Muhammad Majid Aziz, Rabeel Khan, Khalid Mahmood, Nasira Saif-ur-Rehman, Che Suraya Zin

**Affiliations:** 1 Department of Pharmacy Practice, Lahore Medical & Dental College, University of Health Sciences, Lahore 54600, Pakistan; asma.sarwar@lmdc.edu.pk (A.S.K.); taheerzahid21@gmail.com (T.Z.); bariraaijazz@gmail.com (U.e.B.I.); majid.aziz@lmdc.edu.pk (M.M.A.); rabeel.khan74@gmail.com (R.K.); 2 Institute of Information Management, University of the Punjab, Lahore 54000, Pakistan; khalid.im@pu.edu.pk; 3 Kulliyyah of Pharmacy, International Islamic University Malaysia, Kuantan 25200, Malaysia

**Keywords:** antibiotic stewardship, antimicrobial stewardship, hospitals, tertiary care, Pakistan, core elements, CDC, health policy, rational drug use, AMR, LMIC, resistance

## Abstract

**Background:** To restrain antibiotic resistance, the Centers for Disease Control and Prevention (CDC), United States of America, urges all hospital settings to implement the Core Elements of Hospital Antibiotic Stewardship Programs (CEHASP). However, the concept of hospital-based antibiotic stewardship programs is relatively new in Low- and Middle-Income Countries. **Aim:** To appraise the adherence of the tertiary care hospitals to seven CEHASPs. **Design and Setting:** A cross-sectional study in the tertiary care hospitals in Punjab, Pakistan. **Method:** CEHASP assessment tool, (a checklist) was used to collect data from the eligible hospitals based on purposive sampling. The check list had 19 statements to cover seven CEHASPs: *Hospital Leadership Commitment, Accountability, Pharmacy Expertise, Action (Implement Interventions to Improve Antibiotic Use), Tracking Antibiotic Use and Outcomes, Reporting Antibiotic Use and Outcomes,* and *Education.* For each statement, a response of “YES”, “NO” or “Under Process” constituted a score of 2, 0 and 1, respectively, where the higher the scores the better the adherence. Categorical variables were described through descriptive statistics, while independent *t*-test computed group differences. **Result**: A total of 68 hospitals (*n* = 33 public, *n* = 35 private) participated with a response rate of 79.1%. No hospital demonstrated “Perfect” adherence. Roughly half private (48.6%) and more than half public (54.5%) sector hospitals were “Poor“ in adherence. Based on the mean score, there was no significant difference between the private and the public hospitals in terms of comparison of individual core elements. The two most neglected core elements emerged as top priority area were: *Reporting Antibiotic Use and Outcomes and Tracking Antibiotic Use and Outcomes.* **Conclusion:** The current response of Pakistan to implement hospital-based antibiotic stewardship programs is inadequate. This study points out significant gaps of practice both in public and private tertiary care hospitals. A majority of the core elements of antibiotic stewardship are either absent or ”Under Process”. The deficiency/priority areas mentioned require immediate attention of the concerned stakeholders in Pakistan.

## 1. Background

The struggle for survival is one of the basic instincts of living organisms, including bacteria, who have evolved different mechanisms to resist the lethal actions of antibiotics—once considered “magical bullets”. This situation has led to one of the inescapable crises of 21st century called “antibiotic resistance”, i.e., the situation in which antibiotics would no longer be effective against bacteria. These resistant bacteria make even ordinary infections more difficult to treat and surgical procedure riskier to perform. Centuries of progress in improving health and economy is at stake due to antibiotic resistance [1,2]. If healthcare systems do not implement systematic interventions, by 2050, antibiotic resistance may inflict 10 million deaths per year around the globe and an economic loss of USD 100 trillion [3]. The World Health Organization (WHO) recorded grave concerns on the continuous rife in antibiotic resistance and warned of the possibilities of a “post antibiotic era” [2].

Multiple factors are responsible for accelerating antibiotic resistance, however, there exists a strong correlation between the development of resistance and overuse or over prescribing of antibiotics [4,5]. To optimize the use of antibiotics, the WHO placed the implementation of hospital based antibiotic stewardship programs (ASPs) at the heart of its Global Action Plan to curb antibiotic resistance [6]. Antibiotic Stewardship includes “*coordinated interventions designed to improve and measure the appropriate use of [antibiotic] agents by promoting the selection of the optimal [antibiotic] drug regimen including dosing, duration of therapy, and route of administration*” [7]. Thus, the WHO urges hospitals and other healthcare settings (at all levels, i.e., primary, secondary, and tertiary care) to implement some form of ASPs based on local or international guidelines. Subsequently, many high-income countries shifted the focus of public health policy and implemented ASPs in hospitals and other related settings. At the forefront, for instance, the United States Centers for Diseases Control and Prevention (CDC) chartered the Core Elements of Hospital Antibiotic Stewardship Programs (CEHASP) in 2016, updated in 2019, and demanded its implementation in all hospitals across the country [8].

Antibiotic resistance is no longer only a problem of high-income countries, for instance, five Low- and Middle-Income Countries (LMICs) bear more than half (52%) of the burden of mortalities caused by neonatal sepsis associated with resistant strains of bacteria. In some cases, bacteria are resistant to up to 90% of antibiotics [1,2]. Over prescribing, limited surveillance or regulatory framework, and rampant poor practices of infection prevention and control incite a faster spread of resistant bacteria in LMIC. A recent estimate of the prevalence of antibiotic resistance in different regions has raised many red flags and revealed that Asia is home to 70% of emerging resistance, making this region an important focus of ASPs [9]. However, the concept of hospital-based ASPs has gained recent attention in LMICs, and various governments have recorded commitments to take initiatives to implement ASPs in hospitals. Nevertheless, translating national or local commitment into action represents a formidable implementation challenge [10,11].

Pakistan, a country in South Asia, belongs to the World Bank strata of LMICs with a population 216.5 million. In terms of healthcare infrastructure, Punjab is the most developed province that accommodates more than 65% of the population of Pakistan [1,12]. Healthcare is provided by a rapidly expanding network of private hospitals and heavily subsidized public hospitals where the latter fulfils the healthcare needs of the majority of the population [13]. Antibiotic consumption rate is alarmingly high and reportedly Pakistan has the third highest antibiotic consumption in LMICs [14]. Recent point prevalence surveys on antibiotic use in hospitals of Punjab also depict a gloomy situation where patients receive unnecessary antibiotics [15,16]. Furthermore, various studies have reported pervasive infections caused by multidrug resistant and extensively drug resistant bacteria [1]. Rampant inappropriate prescribing requires systematic interventions to change the physician’s attitude, and, in this context, ASPs can play a formidable role to optimize the antibiotic use in hospitals. Pakistan’s National Action Plan (2017) recognizes antibiotic resistance as a key challenge in Pakistan and urges hospitals nationwide to develop and implement ASPs to curtail the continuous surge of resistant infections [17]. However, to what extent standardized ASPs have been instituted in hospitals remains unknown, and there is a paucity of key implementation data and facts on the roll out of ASPs, especially in tertiary care settings. Hence, this study aims to appraise the current adherence levels of tertiary care hospitals to the CEHASPs. It also implies underlying subgroup analysis to identify differences in adherence to CEHASPs in private and public hospitals. The evidence base generated may potentially contribute to the formulation of national or local policies to implement ASPs in hospitals with a prime focus on the gaps and priority areas identified in this study.

## 2. Methods

### 2.1. Study Design, Survey Instrument and Setting

This was a cross-sectional study to collect hospitals’ data on the validated CDC “Antibiotic stewardship program assessment tool” [18]. Compared with other ASP assessment tools, the CDC assessment tool offers a valuable advantage, i.e., a universal applicability, as it can be used to evaluate any setting (resource rich or resource poor). The survey instrument comprised of two parts.

Part one enquired about the demographic details of the hospital, such as name, ownership (public/private), name of the administrative division of Punjab in which the hospital was located, and designation and sign and stamp of the hospital representative providing the information. Part two was comprised of the CDC assessment tool enlisting seven core elements of hospital ASPs. A core element was a broad category of action or strategy that covered a main aspect of antibiotic stewardship (e.g., commitment or education). The seven core elements are: *Hospital Leadership Commitment, Accountability, Pharmacy Expertise, Action (Implement Interventions to Improve Antibiotic Use), Tracking Antibiotic Use and Outcomes, Reporting Antibiotic Use and Outcomes,* and *Education*. Generally, three to four statements (items) covered a unique core element. On a summative scale, for each statement of a core element, a response of “YES”, “NO” or “Under Process” constituted a score of 2, 0 and 1, respectively. There were a total 19 statements covering the seven core elements of hospital ASP (please see the Survey Instrument CDC assessment tool for core elements of hospital antibiotic stewardship programs in Table S1 in Supplementary Materials). Thus, a maximum score of 38 could be possible for a given hospital where the higher the scores the better the adherence. Furthermore, as the seven core elements had a different number of sub-statements, to normalize the data, we used mean in order to compare the individual core element in a standardized way.

A list of tertiary care hospitals was obtained from the Specialized Healthcare & Medical Education Department, government of Punjab, and hospitals were recruited based on the purposive sampling.

### 2.2. Data Collection

Data were collected in a personal meeting with the representative of the administration of the hospitals willing to participate in the survey from 3 December 2019 to 15 January 2020. Any of the following representatives of the hospital administration was eligible to fill the survey: Medical Superintendent, Deputy Medical Superintendent, Assistant Medical Superintendent, Manager/Head of Pharmacy, or Chief Pharmacist. These representatives were chosen because all of them belong to hospital administration, hence, are better aware of the policies and practices in a given hospital. Furthermore, the outcome (survey responses) could not change with the change of the designation, as all are part of the administration team in a hierarchy. The team of data collectors—11 Pharm-D final year students—aimed to reach tertiary care hospitals located in all the nine administrative divisions of the Punjab, and Islamabad, the capital territory. The data collection team remained present throughout the meeting for any query related to the CEHASP. As compared to a survey sent through mail or email, the in-person, self-administration of the survey was opted to ensure quick and high response rate, and an error-free recording of the responses. Final analysis added only those responses which were signed and stamped by any of the earlier mentioned representatives.

### 2.3. Inclusion and Exclusion Criteria

All tertiary care hospitals (both in the public and private sector) located in Punjab and Islamabad were eligible to participate in the survey. For this study, a tertiary care hospital was defined as any hospital enlisted by the Punjab government as an access point for tertiary care, or any teaching or non-teaching hospital in the private or public sector with distinct specialties and facilities, such as ICU, CCU, dialysis, ventilators, in-house lab testing and pharmacy services, etc., and general specialties like surgery, medicine, orthopedics, pediatrics, and gynecology.

Primary care facilities and small secondary care hospitals (<30 beds) were excluded.

### 2.4. Data Management and Statistical Analysis

Statistical Package for Social Sciences (SPSS) (version 22 IBM, Armonk, NY,, USA) was used to manage and analyze data. Descriptive statistics were used to evaluate the extent of adherence, while mean difference between private and public hospitals was analyzed with the help of *t*-tests for each of the core elements mentioned earlier with a *p* value < 0.05 considered significant.

Here, an important point to mention was about the nature of variables. For a particular core element, the score of 2, 1 or 0 against “YES”, “Under Process”, and “NO”, respectively, was taken as numerical variable because the scores were given as real numbers similar to scores in an exam where one point is given on a correct answer leading to a total score. This type of data is not dichotomous and hence may not be confused just because of ”YES/NO”. Thus, mean scores would be based on a summative scale, if choose to answer “YES” for all the statements of the survey, a hospital could achieve a maximum score of 38. The mean scores of hospitals would be related to adherence where the higher the score, the better the adherence to the CEHASP. To assess level of adherence of participating tertiary care hospitals, the total score obtained by the hospitals was assigned a category, for instance, a total score range of 0–11.9, 12–23.9, 24–34.9, and 35–38 was defined as “Poor”, “Moderate”, “Good”, and “Perfect”, respectively.

### 2.5. Ethics

The ethical approval was granted by the Research Ethics Committee, Lahore Pharmacy College, Lahore Medical & Dental College (ref# ETH/LPC/10/08/19). Before taking consent, project information was shared with the representative of all participating hospitals to detail the background, aims, and how the data would be utilized. To ensure the anonymity of hospitals, an individual identification number was allocated to each participating hospital. Data were stored in a password protected computer.

## 3. Results

A total of *n* = 68 hospitals (*n* = 33 public, *n* = 35 private) participated with a response rate of 79.1% (12 private and 6 public hospitals declined due to lack of willingness to share data). Mainly, the respondents were Chief Pharmacist/Director of Pharmacy Services/Manager of Pharmacy Services (36.8%, *n* = 25), followed by Medical Superintendents (29.3%, *n* = 20), Assistant Medical Superintendents (22%, *n* = 15), and Deputy Medical Superintendents (11.8%, *n* = 8).

**Figure 1:**
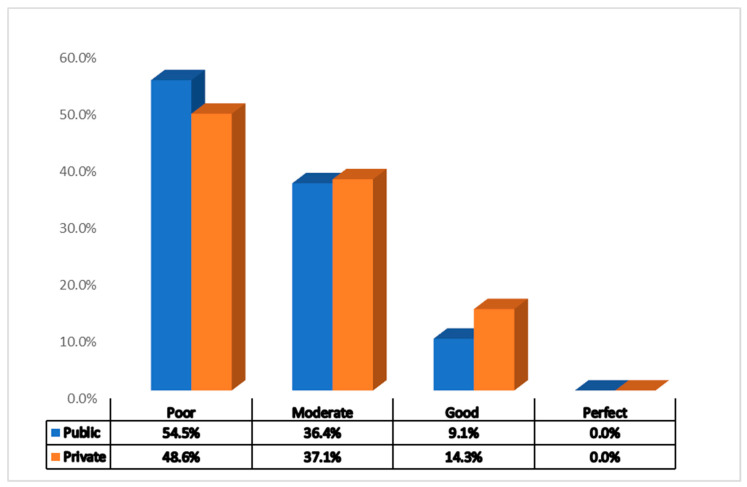
Percentage of public and private tertiary care hospitals in each defined category of adherence for core elements.

The survey reached to diversely located hospitals in all official administrative divisions of Punjab, i.e., Lahore, Rawalpindi, Dera Ghazi Khan, Bahawalpur, Sahiwal, Sargodha Faisalabad, Multan, and Gujranwala, and additionally, the Islamabad Capital Territory (the federal capital), and three other cities in Punjab, Sialkot, Sheikhupura, and Rahim Yar Khan (Table 1). No public or private hospital was in “perfect” compliance with all the seven core elements (Figure 1). The majority of the hospitals, in both the private and public sector, were poorly adherent to the CEHASP. In subgroup analysis, among the public hospitals, only 9.4% hospitals scored high enough to fall in the “Good” category of adherence, while more than half (54.5%) were in the “Poor” category, as depicted in the Figure 1. The situation was more or less the same in the private sector. Among the private hospitals, roughly half (48.6%) were in the “Poor” category, while 14.3% hospital were in the “Good” category of adherence. Furthermore, private and public hospitals had no significant difference in mean scores for adherence to the CEHASP.

In the context of individual core elements, a major lacking was observed in the core element *Reporting Antibiotic Use and Outcomes,* that received the lowest mean in both private and public hospitals, followed by *Tracking Antibiotic Use and Outcomes*.

In subgroup analysis, comparatively, private hospitals performed well (higher mean score) in almost all core elements, however, the difference was not statistically significant as mentioned in Table 2. Nevertheless, public hospitals scored higher in *Tracking Antibiotic Use and Outcomes* (but the difference was not significant, Table 2).

Item level analysis and the scores of the individual statements of each core element in the survey can be found in the Table 3, while corresponding SPSS data is provided in Table S2 of the Supplementary Materials.

**Figure 2:**
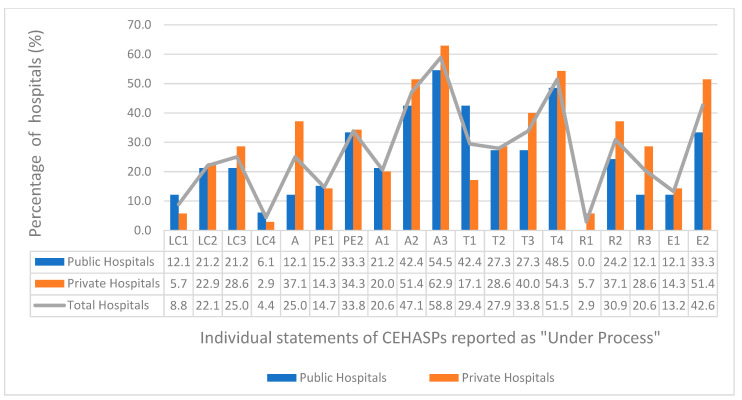
Percentage of hospitals which reported “Under Process” interventions for individual statements of each core element. LC1 to LC4 = statements 1 to 4 of the core element *Hospital Leadership and Commitment.* A = statement of the core element *Accountability.* PE1 to PE2 = statements 1 to 2 of the core element *Pharmacy Expertise.* A1 to A3 = statements 1 to 3 of the core element *Action.* T1 to T4 = statements 1 to 4 of the core element *Tracking Antibiotics Use and Outcomes.* R1 to R3 = statements 1 to 3 of the core element *Reporting Antibiotics Use and Outcomes.* E1 to E2 = statements 1 to 2 of the core element *Education*.

Precisely, one of the statements that covered the core element of Action, “Does your facility have facility-specific treatment recommendations, based on national guidelines and local pathogen susceptibilities, to assist with antibiotic selection for common clinical conditions?”, was reported as the most “Under Process” intervention to improve stewardship in more than half (58.8%) of all the participating hospitals (54.5% public, 62.9% private). It was followed by the statement that covered the core element of Tracking of Antibiotic Use and Outcomes: “Does your stewardship program monitor adherence to facility- specific treatment recommendations?”. It received the status of “Under Process” by 51.5% of the participating hospitals (48.5% public, 54.3% of private). This trend is depicted in the Figure 2.

## 4. Discussion

This study explored the current state of implementation and roll out of ASPs in the tertiary care hospitals of Punjab, Pakistan. Tertiary care represents an advance setting, however, given the findings, unfortunately the majority of the CEHASPs are yet to be implemented in the hospitals. For instance, *Reporting* and *Tracking of Antibiotic Use and Outcomes* are at the heart of any ASPs, however, emerged as the top two most neglected core elements both in private and public hospitals that participated in this study. A possible reason of this implementation failure could be the general absence of a compelling narrative to link the ASPs as the core national health priority to convince the political will. Similarly, response on the four statements that cover *Tracking Antibiotic Use and Outcomes* revealed that preauthorization and prospective audits were evidently absent in an overwhelming majority of the hospitals, especially in private sector. Furthermore, hospitals do not track and document antibiotic use to submit to a provincial/national or international database. Many studies have reported the impact of prospective audits and formular as being most effective measurer [19]. Nevertheless, these two core elements implied two major gaps in practice for any future intervention and may be taken as a top priority. Similarly, *Hospital Leadership Commitment* was notably lacking and indicated another gap in practice. Leadership commitment in a hospital is actually a direct function of the extent of the implementation of ASPs. *Hospital Leadership Commitment* was evaluated through four statements, however, data reveal that not all statements for this core element received high scores. The most underscored statement among the four was, “Does facility leadership provide stewardship program leader(s) with resources (e.g., IT support, training) to effectively operate the program?”. This highlights the lack of resources hospitals have in this part of the world.

Two encouraging findings of this study are related to the two core elements *Accountability* and *Education* of the prescribers, which received the highest mean score in both private and public hospitals. Literature also supports that a small investment on these basic core elements can substantially improve the outcome [20]. The next logical step is to translate commitment into action. Likewise, *Education* of the prescriber and other related healthcare professionals received the second highest score in the CEHASPs, particularly in private sector. It is a positive development as compared to previously reported studies and indicates that more hospitals are providing continuous education to healthcare professionals and are making them accountable for stewardship interventions and programs [21]. Be that as it may, though continuous education seems to be part of practice in the hospitals, it should be improved to the next level as it requires the least resources. One way to improve this deficiency is to engage hospital pharmacists in the stewardship teams. The addition of pharmacists in ASPs has shown to improve the outcomes of antibiotic stewardship interventions, and a recent example may be cited from Africa [9,22]. This is of particular relevance for LMICs like Pakistan, where only a few physicians are available for a huge population of patients, and therefore dissemination of a meaningful education to patients is often compromised. Pakistan also needs to engage its underutilized workforce of pharmacists in hospitals for the same purpose [12,16]. Thus, utilizing pharmacy expertise in antibiotic stewardship activities beyond simple dispensing and procurement is a need in Pakistan. Survey results on utilization and training of hospital pharmacist expertise show moderate progress in adoption of pharmacist expertise in tertiary care hospitals.

Finally, a promising finding of the study is related to those core elements which were reported “Under Process”, i.e., not implemented at the time of study. For instance, more than half of tertiary care hospitals are in the process of implementing treatment guidelines for local susceptible pathogens, as well as tracking the antibiotic use in wards as shown in Figure 2. Furthermore, 33.8% of the participating hospitals are in the process of training and involving pharmacists in ASPs. This shows that an increasing number of hospitals (especially in the private sector) have been sensitized on the issue at hand, and one may expect an action for the implementation soon.

### 4.1. Comparison with the Existing Literature

The findings of this study are in agreement with the results of a previous similar study conducted in outpatient settings of tertiary care hospitals in Punjab [23]. *Reporting Antibiotic Use and Outcomes* *and Tracking Antibiotic Use and Outcomes* remain the top deficiency areas reported in both studies. This state of affairs indicates a lack of action and too-slow implementation process, hence, requires immediate attention of the health authorities.

Our study results may be broadly comparable to the findings of a qualitative study that explored physicians’ views for implementation of the ASPs in public hospitals of Punjab and mentioned that physicians endorsed the implementation of stricter policies on antibiotics surveillance [19]. This is in line with our results of the core element that judged *Accountability to* implement ASPs and received one of the highest scores among the seven CEHASPs. At the same time, our data contradict the findings of the same study that poor familiarity of ASPs exists among the physicians in the hospitals surveyed. One possible reason could be the differences in the sample and data collection time of the two studies. The mentioned study had participating physicians mainly from the public hospitals, while our study included physicians (MS/AMS/DMS) and pharmacists from both private and public sector hospitals in proportion in 2020. Another study conducted in Karachi, a city in the province of Sindh, Pakistan, identified similar gaps in practice of antibiotic stewardship in hospitals [24]. This study reported a serious lack of leadership commitment and reporting antibiotic use in the 44 hospitals that participated, which is in line with our results. However, our study took a step further and reported new findings on various ASPs which were “Under Process” in different hospitals. Furthermore, comparing these two studies, our study findings were based on a diverse sample of the hospitals and thus reported more reliable data. Nevertheless, both studies point out serious lacking in two different provinces of Pakistan (Punjab and Sindh) and collectively portrays a general absence of policies in place.

Compared to other LMICs internationally, the findings of this study imply a poor to moderate adoption and implementation of the core elements of ASPs in hospitals. Substantial progress has been reported, for instance, in African regions where Antibiotic Surveillance and Stewardship Programs have been implemented in a majority of the hospitals and routine surveillance and audits are being performed. These programs have resulted in improving the prescribing habits of not only physicians, but surgeons as well, and improved awareness of the public and professionals. With the inclusion of hospital pharmacists in the stewardship team to lead the stewardship activities, significant reductions in the misuse of antibiotics have been observed [22,25,26]. Similarly, another study conducted in Jordan also aimed to evaluate the adherence of hospitals to the core elements of antibiotic stewardship and reported slow adoption and poor implementation of interventions in the country [27]. However, the data was obtained in 2017 and there could have been improvements in stewardship activities in the meantime.

In the region, Pakistan can learn some lessons from China, which has achieved substantial success in implementation of ASPs in the tertiary care setting, especially in terms of Leadership Commitment and Tracking Antibiotic Use. Data of 116 hospitals from all provinces and municipalities revealed that 94% of the hospitals had formal teams for antibiotic stewardship interventions which ensure tracking of antibiotics use through preauthorization and post prescription reviews with feedback [28]. Thus, in Asia, many countries have started implementing various core elements of hospital-based ASPs, however, in Pakistan the pace is comparatively slow.

### 4.2. Implications for Policy and Practice and Further Research

Periodic cross-sectional audits are mandatory to keep a check on the progress of the implementation of the stewardship activities in hospitals. The results of this study warrant more in-depth quantitative research into core elements that scored low in order to fully understand the quality of antibiotic stewardship in the tertiary care setting. Pakistan has partnered with a US program, Global Antimicrobial Resistance Partnership, and made commitments to curb antibiotic resistance [29]. It is also in parallel to Pakistan’s first National Action Plan (2017) that aims to rationalize the use of antibiotics with a prime focus on ASPs in hospitals. However, the findings of this study voice apprehensions over the implementation failure of translating political commitment into action. Thus, our findings serve as a reminder to expediate the process of the implementation of ASPs in healthcare settings. The gaps identified in this study are more relevant as a broader outline to refine the plan into action. Based on the findings, the current study suggests following implications for policy, practice and further research:

(a) The deficiency areas highlighted in this study, such as the core elements of *Tracking and Reporting of Antibiotic Use*, should be prioritized in any future policy shift and must be given due weight.
(b) Concerned ministry should take a step forward and enforce a low hang intervention of tertiary care hospitals. Here, the concept of low hang intervention refers to interventions that require least resources but yield high outcomes, for instance, *Education* of the prescriber is directly correlated with the improved prescribing habits and patient education.
(c) Hospital pharmacists should be engaged in antibiotic stewardship activities in the hospitals, beyond merely dispensing or procurement of medicines. As of now pharmacy expertise is an official requirement in the CDC proffered CEHASP.
(d) Similar studies need to be conducted in other provinces of Pakistan to draw a holistic picture of the situation to keep a check on the progress of implementation of the ASPs.

### 4.3. Limitations and Strength of the Study

We noted the following limitations and strengths of the study. We only covered tertiary care hospitals in Punjab and ICT, hence, results may be interrupted with caution in terms of generalizability to other provinces of Pakistan. The sample of tertiary care hospitals was, though diverse and large, still purposive, and the participation was voluntary and not an obligation as the case of government funded audits. We did not collect detailed demographic data of the hospitals, such as the number of beds, wards, lab facilities, etc. However, this kind of data were of lesser significance and unrelated to the objectives set in this research.

To the best of our knowledge, this is the first study that extensively covered tertiary care hospitals in Punjab and the Islamabad Capital Territory to appraise the rollout of some form of ASP against a standard, i.e., the CEHASP in Punjab. Additionally, our study aimed to minimize recruitment bias as the data collectors did not opt for a convenient sample of the hospitals in Lahore only, but rather to reach out to hospitals diversely located in different divisions of Punjab. In terms of impact, the study reported specific gaps in ASPs in hospitals in Punjab for future policy. These findings are equally useful for other LMICs from an introspective point of view and urge healthcare stakeholders to encourage hospitals to implement a minimum set of ASPs.

## 5. Conclusions

The current response of Pakistan to implement hospital-based antibiotic stewardship programs is inadequate. The findings of this study further imply a too-slow implementation and adoption of the stewardship programs in the tertiary care hospitals of the most developed province of Pakistan, i.e., Punjab. Despite the grandiose infrastructure and resources private hospitals have in Pakistan, there was no significant difference between the two groups in terms of adherence. While two core elements indicated an encouraging situation, i.e., *Accountability and Education*, nevertheless, overall findings are disappointing and there is still much more that needs to be done. The majority of the core elements of antibiotic stewardship are either absent or ”Under Process” and indicate significant gaps in the practice of antibiotic stewardship. If left unattended, these gaps will inflict immense harm and ultimately affect the appropriate use of antibiotics in hospitals. The findings of this study further point out some urgent priority areas to focus on for action, such as *Tracking* and *Reporting of Antibiotic Use and Outcomes* in hospitals. These priority areas should be given special emphasis in future policy for hospital-based antibiotic stewardship programs. This study urges relevant stakeholders to expediate the implementation of antibiotic stewardship programs in hospitals to curb the growing antibiotic resistance and optimize antibiotic therapy. A significant impact is only possible if a majority of the hospitals in Pakistan implement uniform stewardship programs. We are running our time out for antibiotics, and if implementation of stewardship interventions is further delayed, the resistant infections will have disastrous impact on the healthcare sector and the country’s economy.

## Figures and Tables

**Table 1:** Number of public and private hospitals that participated from each division of Punjab.

Name of Division	No. of Public Hospitals	No. of Private Hospitals
Lahore	14	12
Islamabad	2	2
Rawalpindi	2	2
Faisalabad	3	3
Gujranwala	2	3
Sialkot	1	2
Sheikhupura	1	1
Sargodha	2	2
Multan	2	3
Bahawalpur	1	1
Rahim Yar Khan	1	1
Dera Ghazi Khan	1	1
Sahiwal	1	2
Total	33	35

**Table 2:** Core elements of hospital antibiotics stewardship.

	Sr.		Mean (SD)	Subgroup Analysis		
				Public Hospitals Mean (SD)	Private Hospitals Mean (SD)	*t* * (*p*-Value)
Core Elements of Antibiotic Stewardship	1.	Hospital Leadership Commitment	0.76 (0.71)	0.74 (0.69)	0.78 (0.73)	−0.21 (0.835)
	2.	Accountability	1.34 (0.80)	1.33 (0.89)	1.34 (0.73)	−0.05 (0.961)
	3.	Pharmacy Expertise	1.10 (0.77)	1.09 (0.75)	1.10 (0.79)	−0.05 (0.962)
	4.	Action: Implement Interventions to Improve Antibiotic Use	0.69 (0.52)	0.66 (0.50)	0.71 (0.54)	−0.45 (0.651)
	5.	Tracking Antibiotic Use and Outcomes	0.44 (0.37)	0.45 (0.36)	0.44 (0.38)	−0.21 (0.834)
	6.	Reporting Antibiotic Use and Outcomes	0.20 (0.30)	0.14 (0.25)	0.26 (0.33)	−1.61 (0.113)
	7.	Education	1.29 (0.73)	1.26 (0.82)	1.33 (0.65)	−0.40 (0.693)
		Total mean score	0.72 (0.39)	0.70 (0.41)	0.74 (0.38)	−0.42 (0.678)

Notes: * Independent *t*-test. Abbreviation: SD, standard deviation.

**Table 3:** Item level analysis of core elements of antibiotics stewardship programs.

	Statements	Mean (SD)	Subgroup Analysis		
			Public Hospitals Mean	Private Hospitals Mean	*t* (*p*-Value)
Hospital Leadership Commitment	Does facility leadership provide stewardship program leader(s) dedicated time to manage the program and conduct daily stewardship interventions?	1.00 (0.96)	1.03 (0.95)	0.97 (0.99)	0.25 (0.362)
	Does facility leadership provide stewardship program leader(s) with resources (e.g., IT support, training) to effectively operate the program?	0.31 (0.55)	0.27 (0.52)	0.34 (0.59)	−0.52 (0.315)
	Does your antibiotic stewardship program have a senior executive that serves as a point of contact or “champion” to help ensure the program has resources and support to accomplish its mission?	1.13 (0.86)	1.12 (0.89)	1.14 (0.85)	−0.10 (0.496)
	Do stewardship program leader(s) have regularly scheduled meetings with facility leadership and/or the hospital board to report and discuss stewardship activities, resources and outcomes?	0.60 (0.90)	0.55 (0.87)	0.66 (0.94)	−0.51 (0.250)
Accountability	Does your facility have a leader or co-leaders responsible for program management and outcomes of stewardship activities?	1.34 (0.80)	1.33 (0.89)	1.34 (0.73)	−0.05 (0.041)
Pharmacy Expertise	Does your facility have a pharmacist(s) responsible for leading implementation efforts to improve antibiotic use?	1.35 (0.86)	1.36 (0.86)	1.34 (0.88)	0.10 (0.840)
	Does your pharmacist(s) leading implementation efforts have specific training and/or experience in antibiotic stewardship?	0.84 (0.80)	0.82 (0.81)	0.86 (0.81)	−0.198 (0.931)
Action: Implement Interventions to Improve Antibiotic Use	Does your facility perform prospective audit and feedback for specific antibiotic agents?	0.88 (0.89)	0.88 (0.89)	0.89 (0.90)	−0.03 (0.904)
	Does your facility perform preauthorization for specific antibiotic agents?	0.56 (0.58)	0.55 (0.62)	0.57 (0.56)	−0.18 (0.430)
	Does your facility have facility-specific treatment recommendations, based on national guidelines and local pathogen susceptibilities, to assist with antibiotic selection for common clinical conditions?	0.62 (0.52)	0.55 (0.51)	0.69 (0.53)	−1.12 (0.532)
Tracking Antibiotic Use and Outcomes	Does your antibiotic stewardship program track antibiotic use by submitting to the national/provisional/international center Antimicrobial Use (AU) Option?	0.29 (0.46)	0.42 (0.50)	0.17 (0.38)	2.33 (0.000)
	Does your antibiotic stewardship program monitor prospective audit and feedback interventions by tracking the types of interventions and acceptance of recommendations?	0.54 (0.72)	0.58 (0.75)	0.51 (0.70)	0.35 (0.563)
	Does your antibiotic stewardship program monitor preauthorization interventions by tracking which agents are being requested for which conditions?	0.40 (0.55)	0.33 (0.54)	0.46 (0.56)	−0.93 (0.294)
	Does your stewardship program monitor adherence to facility-specific treatment recommendations?	0.54 (0.53)	0.48 (0.51)	0.60 (0.55)	−0.89 (0.65)
Reporting Antibiotic Use and Outcomes	Does your antibiotic stewardship program share facility and/or individual prescriber-specific reports on antibiotic use with prescribers?	0.03 (0.17)	0.00 (0.00)	0.06 (0.24)	−1.40 (0.004)
	Does your antibiotic stewardship program report adherence to treatment recommendations to prescribers (e.g., results from medication use evaluations, etc.)?	0.31 (0.47)	0.24 (0.44)	0.37 (0.49)	−1.15 (0.026)
	Has your facility distributed a current antibiogram to prescribers?	0.26 (0.51)	0.18 (0.47)	0.34 (0.54)	−1.32 (0.029)
Education	Does your stewardship program provide education to prescribers and other relevant staff on optimal prescribing, adverse reactions from antibiotics, and antibiotic resistance?	1.49 (0.82)	0.33 (0.89)	1.57 (0.74)	−1.20 (0.035)
	Does your stewardship program provide education to prescribers as part of the prospective audit and feedback process (sometimes called “handshake stewardship”)?	1.13 (0.75)	1.18 (0.81)	1.09 (0.70)	0.52 (0.104)

## Data Availability

All the data have been provided in the supplementary information.

## Supplementary Materials

The following are available online at https://www.mdpi.com/article/10.3390/antibiotics10080906/s1, Table S1: Survey Instrument CDC assessment tool for core elements of hospital antibiotic stewardship programs; Table S2: Individual hospital response SPSS file.

## References

[1] Bilal H., Khan M.N., Rehman T., Hameed M.F., Yang X. (2021). Antibiotic resistance in Pakistan: A systematic review of past decade. BMC Infect. Dis..

[2] WHO (2019). No time to wait: Securing the future from drug-resistant infections. Interagency Coordination Group on Antimicrobial Resistance.

[3] Dadgostar P. (2019). Antimicrobial Resistance: Implications and Costs. Infect. Drug Resist..

[4] Aslam B., Wang W., Arshad M.I., Khurshid M., Muzammil S., Rasool M.H., Nisar M.A., Alvi R.F., Aslam M.A., Qamar M.U. (2018). Antibiotic resistance: A rundown of a global crisis. Infect. Drug Resist..

[5] Blaskovich M.A.T. (2018). The Fight Against Antimicrobial Resistance Is Confounded by a Global Increase in Antibiotic Usage. ACS Infect. Dis..

[6] World Health Organization (2015). Global Action Plan on Antimicrobial Resistance.

[7] Fishman N., Society for Healthcare Epidemiology of America, Infectious Diseases Society of America, Pediatric Infectious Diseases Society (2012). Policy Statement on Antimicrobial Stewardship by the Society for Healthcare Epidemiology of America (SHEA), the Infectious Diseases Society of America (IDSA), and the Pediatric Infectious Diseases Society (PIDS). Infect. Control Hosp. Epidemiol..

[8] CDC (2019). The Core Elements of Hospital Antibiotic Stewardship Programs, 23.

[9] Gebretekle G.B., Mariam D.H., Abebe W., Amogne W., Tenna A., Fenta T.G., Libman M., Yansouni C.P., Semret M. (2018). Opportunities and barriers to implementing antibiotic stewardship in low and middle-income countries: Lessons from a mixed-methods study in a tertiary care hospital in Ethiopia. PLoS ONE.

[10] Saleem A.F., Pethani A. (2020). Antimicrobial Stewardship—Do we need it in Pakistan?. JPMA J. Pak. Med. Assoc..

[11] Cox J.A., Vlieghe E., Mendelson M., Wertheim H., Ndegwa L., Villegas M.V., Gould I., Hara G.L. (2017). Antibiotic stewardship in low- and middle-income countries: The same but different?. Clin. Microbiol. Infect..

[12] Atif M., Ihsan B., Malik I., Ahmad N., Saleem Z., Sehar A., Babar Z.-U.-D. (2021). Antibiotic stewardship program in Pakistan: A multicenter qualitative study exploring medical doctors’ knowledge, perception and practices. BMC Infect. Dis..

[13] Hayat K., Rosenthal M., Gillani A.H., Zhai P., Aziz M.M., Ji W., Chang J., Hu H., Fang Y. (2019). Perspective of Pakistani Physicians towards Hospital Antimicrobial Stewardship Programs: A Multisite Exploratory Qualitative Study. Int. J. Environ. Res. Public Health.

[14] Klein E.Y., Van Boeckel T.P., Martinez E.M., Pant S., Gandra S., Levin S.A., Goossens H., Laxminarayan R. (2018). Global increase and geographic convergence in antibiotic consumption between 2000 and 2015. Proc. Natl. Acad. Sci. USA.

[15] Mubarak N. (2021). A multicentre point prevalence survey of the antibiotic use in tertiary care hospitals in Punjab. Antibiotic.

[16] Saleem Z., Hassali M.A., Versporten A., Godman B., Hashmi F.K., Goossens H., Saleem F. (2019). A multicenter point prevalence survey of antibiotic use in Punjab, Pakistan: Findings and implications. Expert Rev. Anti-Infective Ther..

[17] MoNHSRC (2017). Antimicrobial Resistance National Action Plan, Pakistan.

[18] CDC Assessment Tool (2019). The Core Elements of Hospital Antibiotic Stewardship Programs. Antibiotic Stewardship Program Assessment Tool.

[19] Hayat K., Rosenthal M., Zhu S., Gillani A.H., Chang J., Bogale A.A., Kabba J.A., Yang C., Jiang M., Zhao M. (2019). Attitude of clinicians towards hospital-based antimicrobial stewardship programs: A multicenter cross-sectional study from Punjab, Pakistan. Expert Rev. Anti-Infect. Ther..

[20] McKnight J., Maina M., Zosi M., Kimemia G., Onyango T., Schultsz C., English M., Tosas-Auguet O. (2019). Evaluating hospital performance in antibiotic stewardship to guide action at national and local levels in a lower-middle income setting. Glob. Health Action.

[21] Atif M., Asghar S., Mushtaq I., Malik I., Amin A., Babar Z.-U.-D., Scahill S. (2019). What drives inappropriate use of antibiotics? A mixed methods study from Bahawalpur, Pakistan. Infect. Drug Resist..

[22] Gebretekle G.B., Mariam D.H., Taye W.A., Fentie A.M., Degu W.A., Alemayehu T., Beyene T., Libman M., Fenta T.G., Yansouni C.P. (2020). Half of Prescribed Antibiotics Are Not Needed: A Pharmacist-Led Antimicrobial Stewardship Intervention and Clinical Outcomes in a Referral Hospital in Ethiopia. Front. Public Health.

[23] Raheem M., Anwaar S., Aziz Z., Raja S.A., Saif-Ur-Rehman N., Mubarak N. (2020). Adherence to the Core Elements of Outpatient Antibiotic Stewardship: A Cross-Sectional Survey in the Tertiary Care Hospitals of Punjab, Pakistan. Infect. Drug Resist..

[24] Mushtaque M., Khalid F., Ishaqui A.A., Masood R., Maqsood M.B., Muhammad I.N. (2019). Hospital Antibiotic Stewardship Programs—Qualitative analysis of numerous hospitals in a developing country. Infect. Prev. Pract..

[25] Gitaka J., Kamita M., Mureithi D., Ndegwa D., Masika M., Omuse G., Ngari M., Makokha F., Mwaura P., Mathai R. (2020). Combating antibiotic resistance using guidelines and enhanced stewardship in Kenya: A protocol for an implementation science approach. BMJ Open.

[26] Sartelli M., Hardcastle T.C., Catena F., Chichom-Mefire A., Coccolini F., Dhingra S., Haque M., Hodonou A., Iskandar K., Labricciosa F.M. (2020). Antibiotic Use in Low and Middle-Income Countries and the Challenges of Antimicrobial Resistance in Surgery. Antibiotics.

[27] Ababneh M.A., Issa N., Alkhatatbeh M. (2017). Evaluation of core elements of antimicrobial stewardship programs in Jordanian hospitals. Jordan J. Pharm. Sci..

[28] Zhou J., Ma X. (2019). A survey on antimicrobial stewardship in 116 tertiary hospitals in China. Clin. Microbiol. Infect..

[29] Khan E.A. (2018). Situation Analysis Report on Antimicrobial Resistance in Pakistan—Findings and Recommendations for Antibiotic Use and Resistance.

