# Supplementary material for: Assessment of Adherence to the Core Elements of Hospital Antibiotic Stewardship Programs: A Survey of the Tertiary Care Hospitals in Punjab, Pakistan
Source: Antibiotics (Basel). 2021 Jul 24;10(8):906. doi: 10.3390/antibiotics10080906 (PMC8388777; doi:10.3390/antibiotics10080906)
Supplement: Supplementary file 1 [file antibiotics-10-00906-s001.zip › antibiotics-1261107-supplementary.pdf]

Supplementary Materials

Table S1. Survey Instrument CDC assessment tool for core elements of hospital antibiotic stewardship programs
Table S2. Individual hospital response SPSS file

Table S1. Survey Instrument CDC assessment tool for core elements of hospital antibiotic stewardship programs

CORE ELEMENTS OF HOSPITAL ANTIBIOTIC STEWARDSHIP PROGRAMS: ASSESSMENT TOOL		ESTABLISHED AT FACILITY
**Hospital Leadership Commitment**	1. [Priority Example] Does facility leadership provide stewardship program leader(s) dedicated time to manage the program and conduct daily stewardship interventions?	Yes/No/Under Process
	2. [Priority Example] Does facility leadership provide stewardship program leader(s) with resources (e.g, IT support, training) to effectively operate the program?	Yes/No/Under Process
	3. [Priority Example] Does your antibiotic stewardship program have a senior executive that serves as a point of contact or "champion" to help ensure the program has resources and support to accomplish its mission?	Yes/No/Under Process
	4. [Priority Example] Do stewardship program leader(s) have regularly scheduled meetings with facility leadership and/or the hospital board to report and discuss stewardship activities, resources and outcomes?	Yes/No/Under Process
**Accountability**	1. Does your facility have a leader or co-leaders responsible for program management and outcomes of stewardship activities?	Yes/No/Under Process
**Pharmacy Expertise**	1. Does your facility have a pharmacist(s) responsible for leading implementation efforts to improve antibiotic use?	Yes/No/Under Process
	2. Does your pharmacist(s) leading implementation efforts have specific training and/or experience in antibiotic stewardship?	Yes/No/Under Process
**Action: Implement Interventions to Improve Antibiotic Use**	1. [Priority Example] Does your facility perform prospective audit and feedback for specific antibiotic agents?	Yes/No/Under Process
	2. [Priority Example] Does your facility perform preauthorization for specific antibiotic agents?	Yes/No/Under Process
	3. [Priority Example] Does your facility have facility-specific treatment recommendations, based on national guidelines and local pathogen susceptibilities, to assist with antibiotic selection for common clinical conditions?	Yes/No/Under Process

**Tracking Antibiotic Use and Outcomes**	1. [Priority Example] Does your antibiotic stewardship program track antibiotic use by submitting to the national/provisional/international center Antimicrobial Use (AU) Option?	Yes/No/Under Process
	2. [Priority Example] Does your antibiotic stewardship program monitor prospective audit and feedback interventions by tracking the types of interventions and acceptance of recommendations?	Yes/No/Under Process
	3. [Priority Example] Does your antibiotic stewardship program monitor preauthorization interventions by tracking which agents are being requested for which conditions?	Yes/No/Under Process
	4. [Priority Example] Does your stewardship program monitor adherence to facility- specific treatment recommendations?	Yes/No/Under Process
**Reporting Antibiotic Use and Outcomes**	1. Does your antibiotic stewardship program share facility and/or individual prescriber-specific reports on antibiotic use with prescribers?	Yes/No/Under Process
	2. Does your antibiotic stewardship program report adherence to treatment recommendations to prescribers (e.g., results from medication use evaluations, etc)?	Yes/No/Under Process
	3. Has your facility distributed a current antibiogram to prescribers?	Yes/No/Under Process
**Education**	1. Does your stewardship program provide education to prescribers and other relevant staff on optimal prescribing, adverse reactions from antibiotics, and antibiotic resistance?	Yes/No/Under Process
	2. Does your stewardship program provide education to prescribers as part of the prospective audit and feedback process (sometimes called “handshake stewardship”)?	Yes/No/Under Process

Table S2 Individual hospital response SPSS file

ID	Hospital_division	Leadership_com	Leadership_com	Leadership_com	Leadership_com	Accountability	Pharmacy_expe	Pharmacy_expe	Action_1	Action_2	Action_3	Tracking_1	Tracking_2	Tracking_3	Tracking_4	Reporting_1	Reporting_2	Reporting_3	Education_1	Education_2
H1	Public	Yes	No	Yes	Yes	Yes	Yes	Yes	No	Under process	Under process	Under process	No	Under process	Under process	No	Under process	No	Yes	Yes
H2	Private	Yes	No	Yes	Yes	Under process	Yes	Yes	Under process	Under process	Under process	No	Under process	Under process	Under process	No	No	No	Yes	Under process
H3	Public	No	No	No	No	No	Yes	Under process	No	No	No	No	No	No	Under process	No	No	No	Yes	Yes
H4	Public	Under process	Under process	Under process	No	Yes	Yes	Under process	No	No	No	Under process	No	No	No	No	No	No	Yes	Under process
H5	Public	No	No	No	No	No	Yes	No	No	No	No	Under process	No	No	No	No	No	No	No	No
H6	Public	Yes	No	Yes	No	Yes	Yes	No	Yes	No	Under process	No	Under process	No	Under process	No	No	No	Yes	Yes
H7	Private	No	No	Under process	No	No	Yes	Under process	Under process	No	Under process	No	Under process	No	Under process	No	No	No	Yes	Under process
H8	Private	Yes	No	Yes	Yes	Yes	Under process	No	No	No	No	No	No	No	Under process	No	No	No	Yes	No
H9	Public	Yes	No	Yes	Yes	No	Yes	Under process	No	No	Under process	No	No	No	Under process	No	No	No	No	No
H10	Public	Yes	Under process	Yes	Yes	Yes	Yes	Yes	Under process	Under process	Under process	No	Yes	Under process	Under process	No	No	Yes	Yes	Yes
H11	Public	No	No	No	No	Yes	Yes	Under process	Yes	No	Under process	No	Yes	No	Under process	No	No	No	Yes	Yes
H12	Public	Yes	No	Yes	Yes	Yes	Yes	Yes	No	No	Under process	Under process	No	No	Under process	No	No	No	Yes	Yes
H13	Public	No	No	No	No	No	Yes	Under process	No	No	Under process	Under process	No	No	Under process	No	No	No	Yes	Under process
H14	Public	Under process	No	Under process	No	Yes	Yes	Under process	Under process	Yes	No	Under process	No	Yes	Under process	No	Under process	No	Yes	Yes
H15	Private	No	No	Under process	No	Yes	No	No	Yes	Under process	Under process	No	No	No	No	No	No	No	No	No
H16	Public	Under process	No	Under process	No	No	No	Under process	Under process	Under process	Under process	Under process	No	No	Under process	No	No	No	No	No
H17	Private	Under process	Under process	No	No	Under process	Yes	No	No	No	No	No	No	No	No	No	No	No	No	No
H18	Private	Yes	Under process	Yes	Yes	Yes	Yes	Yes	No	Under process	Under process	No	No	Under process	Under process	No	Under process	No	Yes	Under process
H19	Public	Yes	No	Yes	No	Yes	Yes	Under process	No	Under process	Under process	No	No	Under process	No	No	No	No	Yes	Yes
H20	Public	Yes	No	Yes	Under process	Yes	Under process	Under process	Yes	No	No	No	Under process	No	No	No	No	No	Yes	Under process
H21	Private	No	No	Under process	No	Under process	Yes	Under process	Yes	Under process	Under process	Under process	Under process	No	No	No	No	Under process	Under process	No
H22	Public	No	No	No	No	Yes	Under process	No	Yes	No	No	No	No	No	No	No	No	No	Yes	Under process
H23	Private	Yes	No	Yes	Yes	Yes	Yes	Yes	Yes	No	Under process	Under process	Yes	No	Under process	No	Under process	Under process	Yes	Yes
H24	Private	No	No	Under process	No	Yes	No	No	Yes	Under process	Under process	No	No	No	No	No	Under process	Under process	Yes	Yes
H25	Public	No	No	Under process	No	Under process	Yes	Under process	Yes	Under process	No	No	Under process	Under process	No	No	No	No	No	No
H26	Public	No	No	No	No	Yes	Yes	Yes	Under process	Under process	Under process	No	No	No	No	No	No	No	Under process	Under process
H27	Public	Under process	Under process	Under process	No	Yes	No	No	Under process	Under process	Under process	Under process	Under process	No	No	No	No	No	Yes	Yes
H28	Public	No	No	Under process	No	Under process	Yes	No	Yes	Under process	Under process	No	Under process	No	No	No	No	No	No	Under process
H29	Private	Yes	No	Yes	No	Yes	Yes	Under process	Yes	Under process	Under process	No	No	No	No	No	No	No	Yes	Under process
H30	Private	Yes	No	No	No	Yes	No	No	No	No	No	No	No	No	No	No	No	No	Under process	Under process
H31	Private	Yes	Under process	Yes	Under process	Yes	Under process	Under process	Yes	Under process	Under process	No	Yes	Under process	Under process	Under process	Under process	Under process	Yes	Yes
H32	Public	No	No	No	No	No	Under process	No	Under process	Under process	No	Under process	Under process	No	Under process	No	No	No	Under process	Under process
H33	Public	Yes	No	Yes	No	Yes	Yes	Under process	No	Under process	Under process	No	No	Under process	Under process	No	Under process	Under process	Under process	Under process
H34	Public	Yes	Yes	Yes	Yes	Yes	Yes	Yes	Yes	No	Under process	Under process	Yes	No	Under process	No	Under process	No	Yes	Yes
H35	Public	Yes	Under process	Yes	No	Yes	Yes	Yes	Yes	Under process	Under process	Under process	Under process	No	No	No	Under process	Under process	Yes	Yes
H36	Public	Yes	Under process	Yes	Yes	Yes	Yes	Yes	Yes	Yes	Under process	No	Yes	Under process	Under process	No	Under process	Under process	Yes	Yes
H37	Public	Yes	No	Yes	No	Under process	No	No	No	No	No	No	No	No	No	No	No	No	No	No
H38	Private	Yes	Under process	Yes	Yes	Yes	Yes	Yes	Yes	Under process	Under process	No	Under process	Under process	Under process	No	No	No	Yes	Yes
H39	Private	Yes	Yes	Yes	Yes	Yes	Yes	Yes	Yes	Yes	Yes	No	Yes	Yes	Yes	No	Under process	Under process	Yes	Yes
H40	Private	No	No	Under process	No	Yes	Yes	Under process	Under process	Under process	Under process	Under process	No	No	No	Under process	Under process	Under process	No	Under process
H41	Public	No	No	Under process	No	Yes	Under process	No	Under process	Under process	No	Under process	Under process	Under process	No	No	No	No	Under process	Under process
H42	Public	No	No	No	No	Under process	No	No	No	No	No	Under process	No	No	No	No	No	No	Yes	Under process
H43	Private	Yes	Under process	Yes	Yes	Yes	Yes	Yes	Yes	Under process	Under process	Under process	Under process	Under process	Under process	No	Under process	Under process	Yes	Yes
H44	Private	No	No	No	No	No	Yes	Under process	Yes	Under process	Under process	No	Under process	Under process	Under process	No	No	No	No	No
H45	Private	No	No	Under process	No	Yes	No	No	Yes	No	No	No	Yes	No	No	No	No	No	Yes	Yes
H46	Public	Yes	Under process	Yes	Yes	Yes	No	No	Yes	Under process	Under process	No	Under process	Under process	Under process	No	Under process	No	Yes	Yes
H47	Private	Yes	Under process	Yes	Yes	Yes	Yes	Yes	No	Under process	Under process	No	No	Under process	Under process	No	No	No	Yes	Under process
H48	Private	No	No	No	No	Yes	No	No	No	No	No	No	No	No	No	No	No	No	Yes	Under process
H49	Private	Yes	Under process	Yes	No	Under process	Under process	No	No	No	No	No	No	No	No	No	No	No	Yes	Under process
H50	Private	No	No	Under process	No	No	Yes	Under process	No	Under process	Under process	No	No	Under process	Under process	No	No	No	Under process	Under process
H51	Public	No	No	No	No	No	No	No	No	No	No	No	No	No	No	No	No	No	No	No
H52	Private	No	No	Under process	No	Under process	No	No	No	No	Under process	Under process	No	No	Under process	No	No	No	Yes	Under process
H53	Private	Under process	No	Under process	No	Under process	No	No	No	No	Under process	Under process	No	No	Under process	No	No	No	Yes	Under process
H54	Public	Yes	No	Yes	Under process	Yes	Yes	Yes	Yes	Under process	Under process	Under process	Yes	Under process	Under process	No	Under process	Under process	Yes	Yes
H55	Private	No	No	No	No	Under process	Yes	Under process	No	Under process	Under process	No	No	Under process	Under process	No	Under process	No	Under process	Under process
H56	Public	No	No	No	No	Yes	No	No	No	No	No	No	No	No	No	No	No	No	No	No
H57	Private	No	No	Under process	No	No	Under process	No	Under process	Under process	No	No	Under process	Under process	Under process	No	No	No	Under process	No
H58	Private	Yes	No	Yes	Yes	Yes	Yes	Under process	No	Under process	Under process	No	No	Under process	Under process	No	Under process	No	Yes	Yes
H59	Private	No	No	No	No	No	No	No	No	No	No	No	No	No	No	No	No	No	No	No
H60	Private	Yes	Under process	Yes	Yes	Under process	Yes	Yes	Under process	Under process	Under process	No	Under process	Under process	Under process	No	Under process	No	Yes	Under process
H61	Private	Yes	No	Yes	No	Yes	No	No	Yes	No	No	No	Under process	No	No	No	No	Yes	Yes	Yes
H62	Private	No	No	No	No	Under process	Yes	Under process	No	No	No	No	No	No	No	No	Under process	Under process	Yes	Under process
H63	Private	No	No	No	No	Under process	Yes	Under process	No	No	No	No	No	No	No	No	Under process	Under process	Yes	Under process
H64	Private	No	No	No	No	Under process	Yes	Yes	No	No	No	No	No	No	No	No	Under process	Under process	Yes	Under process
H65	Private	Yes	Yes	Yes	Yes	Under process	Under process	No	Under process	No	Under process	No	No	Under process	Under process	No	No	No	Yes	Under process
H66	Private	No	No	No	No	Under process	Yes	Under process	Under process	Under process	Under process	No	Under process	Under process	Under process	No	No	No	Yes	Yes
H67	Public	Yes	Under process	Yes	Yes	No	Under process	No	No	No	No	No	No	No	No	No	No	No	Yes	Under process
H68	Public	No	No	No	No	No	No	No	No	No	No	No	No	No	No	No	No	No	No	No
